# A National Approach to Promoting Health Equity in Cardiovascular Disease Prevention: Implementation Science Strengths, Opportunities, and a Changing Chronic Disease Context

**DOI:** 10.1007/s11121-023-01585-3

**Published:** 2024-01-08

**Authors:** Erika B. Fulmer, Aysha Rasool, Sandra L. Jackson, Marla Vaughan, Feijun Luo

**Affiliations:** 1 Division for Heart Disease and Stroke Prevention, Centers for Disease Control and Prevention, 4770 Buford Highway, Building 107, Atlanta, GA 30341 USA; 2 Oak Ridge Institute for Science and Education, Oak Ridge, TN USA

## Abstract

In the USA, structural racism contributes to higher rates of cardiovascular disease (CVD) including hypertension, heart disease, and stroke among African American persons. Evidence-based interventions (EBIs), which include programs, policies, and practices, can help mitigate health inequities, but have historically been underutilized or misapplied among communities experiencing discrimination and exclusion. This commentary on the special issue of *Prevention Science*, “Advancing the Adaptability of Chronic Disease Prevention and Management Through Implementation Science,” describes the Centers for Disease Control and Prevention, Division for Heart Disease and Stroke Prevention’s (DHDSP’s) efforts to support implementation practice and highlights several studies in the issue that align with DHDSP’s methods and mission. This work includes EBI identification, scale, and spread as well as health services and policy research. We conclude that implementation practice to enhance CVD health equity will require greater coordination with diverse implementation science partners as well as continued innovation and capacity building to ensure meaningful community engagement throughout EBI development, translation, dissemination, and implementation.

## Introduction

In the USA, cardiovascular disease (CVD) is the largest contributor to racial disparities in life expectancy (Purnell et al., 2016). Structural racism—and inequities in the distribution of social, economic, geographic, and environmental conditions that promote health—contributes to higher rates of hypertension, heart disease, and stroke among African American persons (Carnethon et al., 2017; Tsao et al., 2023; Brothers et al., 2019). Public health evidence-based interventions (EBIs), which include programs, policies, and practices, can help mitigate health inequities and address disparities (Ben Charif et al., 2017). However, EBIs have historically been underutilized or misapplied among communities experiencing discrimination and exclusion, because researchers fail to (1) include culturally diverse groups in foundational efficacy and effectiveness studies; (2) consider and mitigate the unique barriers faced by historically marginalized communities; or (3) build upon the inherent strengths of these same communities (Moise et al., 2022).

The Centers for Disease Control and Prevention’s (CDC’s) Division for Heart Disease and Stroke Prevention (DHDSP) focuses on enhancing health equity and mitigating disparities via systems-level strategies to prevent and manage CVD (Centers for Disease Control and Prevention, 2019, 2023a). This work involves applying EBIs that can be tailored, replicated, and scaled to best serve the disease prevention needs of people who are at increased risk for CVD, including historically marginalized communities. Implementation in public health practice occurs via nine DHDSP-funded programs that support state, county, and local health departments; tribal organizations; as well as non-governmental organizations. To achieve its mission, DHDSP applies (1) implementation research, which examines approaches to translating knowledge to best enact public health action, and (2) implementation practice, which applies and adapts these approaches in different contexts (Ramaswamy et al., 2019). DHDSP, in collaboration with the National Network of Public Health Institutes (NNPHI), contributed to this special issue, “Advancing the Adaptability of Chronic Disease Prevention and Management Through Implementation Science.” The special issue highlights innovations in implementation research and practice that will enhance chronic disease prevention and management, and advance health equity. This commentary describes DHDSP’s efforts to support implementation practice and highlights several studies in this issue that align with DHDSP’s methods and mission.

## Applying Implementation Science to Enhance Health Equity in CVD Prevention

### EBI Identification, Scale, and Spread

DHDSP’s *Best Practices Guide for Heart Disease and Stroke: A Guide to Effective Approaches and Strategies* presents EBIs for heart disease and stroke prevention and management. The Best Practices Guide applies a health equity lens to help DHDSP-funded recipients, health care professionals, and public health practitioners better understand and align their implementation efforts (Centers for Disease Control and Prevention, 2022). Toward that end, DHDSP applies methodologies to build practice-based evidence, helping partners to scale and spread EBIs in high-burden health care settings and among populations disproportionately affected by CVD. Such implementation practice methods include systematic screening and evaluability assessments to identify promising practices as well as exploratory, rapid, and effectiveness evaluations to determine core elements of effective implementation and the extent of positive CVD-related outcomes. Once a strategy is found to be effective, DHDSP aims to share each strategy by (1) disseminating it through products and resources to DHDSP-funded recipients, health care professionals, and public health practitioners; (2) using it to inform the work in our funded programs; (3) scaling and spreading the effective strategy in low-resource settings; and (4) evaluating its implementation in new settings or contexts.

Over the past decade, DHDSP’s evaluation team has deployed these methods to build evidence around strategies that enhance CVD prevention. Some newer strategies include (1) engaging community health workers and (2) implementing health-system-wide hypertension management programs, patient-centered medical homes, and programs that address social determinants of health and advance health equity. This approach to implementation allows DHDSP to translate evaluative lessons learned into public health action. In this special issue, Lynch et al. evaluates the acceptability, appropriateness, feasibility, and potential effectiveness of an EBI to lower blood pressure, which was conducted in a trusted Black church setting (Lynch, et al., 2023). These efforts relied on trained community health workers, as well as support from the same church community. The evaluation notes that the unique intervention and setting may widen the outreach to people who are at increased risk for CVD.

For its CVD prevention programs, DHDSP also conducts economic evaluations that examine cost, cost effectiveness, and cost-benefit analyses (Yarnoff et al., 2022; MacLeod et al., 2021). These evaluations guide decision-makers, funded recipients, and partners as they prioritize, scale, and spread EBIs. Additionally, DHDSP applies quasi-experimental methods—including difference in differences, instrumental variable, propensity score matching, and regression discontinuity—to assess health and economic impacts of EBIs among priority populations (Rivera et al., 2022).

DHDSP prioritizes social determinants of health and health equity in CVD prevention and control. Recent research examines the effects of Medicaid expansion and the Affordable Care Act on health equity in CVD, as well as the association between telehealth use and reduction in rural-urban disparities in cardiovascular care access (Ng et al., 2021; Zhang et al., 2018, 2019). Additionally, DHDSP is contributing to CDC’s CORE Health Equity Goals by enhancing access to meaningful metrics related to key determinants of cardiovascular health, including race and ethnicity, gender, education, employment, income, housing, and health insurance (Centers for Disease Control and Prevention, 2023a). The Division is training partners and decision-makers on how to use these data to inform the equitable implementation of EBIs.

Several articles in this special issue evaluated implementation processes and costs of program delivery within historically marginalized communities. For example, Tucker-Brown et al. evaluated implementing a hypertension management program, originally developed in a high-resource health setting, in a multi-site, southeastern Federally Qualified Health Center (FQHC) (Tucker Brown et al., 2023). The team-based, patient-centered program required adaptations to better suit the FQHC setting. The evaluation assessed implementation processes, program costs, facilitators, and barriers.

### Health Services Research

Health services research examines how various factors—population characteristics and behaviors, payment structures, organizations, and health care services and technologies—affect health care access, quality, costs, and outcomes. DHDSP conducts research and surveillance on topics such as implementation of guidelines; gaps in use of evidence-based health services; variation in quality of care across communities, geographic regions, and systems; and disparities, burden, and trends in cardiovascular risk factors and CVD nationally and among priority populations. For instance, DHDSP has created a methodology to conduct surveillance of cardiac rehabilitation. Cardiac rehabilitation is an underutilized, evidence-based secondary prevention program that improves outcomes following a cardiac event or procedure (Centers for Disease Control and Prevention, 2023b; Ades, 2001). Less than one-third of patients who are eligible for cardiac rehab engage in it, and participation is lower among women compared to men, and lower among Hispanic and non-Hispanic Black persons compared to non-Hispanic White persons (Keteyian et al., 2022). Recent research examines geographic variation in cardiac rehabilitation availability (Duncan et al., 2023). In addition to evidence-based clinical services, DHDSP promotes healthy behaviors, including anti-hypertensive medication and/or treatment adherence, and blood pressure self-monitoring (Jackson et al., 2022; Wall et al., 2022).

In this special supplement, Aschbrenner et al. evaluated the feasibility and acceptability of a stakeholder and equity data-driven implementation process, to achieve greater access and equitable outcomes (Aschbrenner et al., 2022). The process used health care data to identify gaps and rapidly adapt colorectal cancer and social needs screening EBIs among patient groups in several FQHCs. In another paper examining disparities within EBIs in safety-net primary care settings, Tsui et al. studied clinic and community member experiences with implementing EBIs for HPV vaccination (Tsui, et al., 2023). Both studies speak to clinical workforce time and resource constraints, as well as the related challenges in prioritizing evidence-based chronic disease care during the COVID-19 pandemic. However, Tsui et al. noted that the pandemic led to innovations in community level vaccine delivery and increased awareness of immunization strategies among policymakers.

### Policy Research

Legal epidemiology is the study of law as a factor in the cause, distribution, and prevention of disease and injury (Thompson et al., 2020). DHDSP’s legal epidemiology portfolio focuses on policy topics regarding the equitable access, reach, uptake, and sustainability of evidence-based strategies for CVD prevention and management, as well as policies impacting social determinants of health. This work is informed by policy research that examines the implementation and outcomes of state law (Fulmer et al., 2020). As part of the policy research continuum, such policy implementation studies assess how supporting structures aid or obstruct the equitable implementation and enforcement of evidence-informed law across differing jurisdictions, settings, and populations (Wennerstrom et al., 2021; Gilchrist et al., 2020; Kulcsar et al., 2014). Public health practitioners use these findings to identify critical implementation actors, methods, and approaches, which then promote the diffusion of public health policy.

To date, DHDSP’s policy implementation studies have engaged an array of partners and subject matter experts throughout project planning, execution, interpretation, and dissemination. However, there is a need to amplify the diverse voices and lived experiences of historically marginalized communities in relation to public health policy. This will require enhanced engagement, equitable evaluative methods, and robust data systems to identify relevant laws’ differential implementation and effects among and across priority populations. As part of this special issue, McGinty et al. examined the complexities of scaling interventions, including an evidence-based integrated care model shown to improve CVD care for people with serious mental illness, through health policy implementation (MgGinty et al., 2023). The paper describes several innovations in policy implementation research.

## Conclusion

DHDSP applies a broad portfolio of work across a variety of setting and populations to support implementation practice and achieve CVD health equity. This portfolio includes the identification, scale, and spread of diverse EBIs which include public health programs, policies, and evidence-based health services. However, advancing health equity in CVD prevention and treatment will require continued innovation and capacity building if we are to ensure the meaningful engagement of communities throughout EBI development, translation, dissemination, implementation—and to support structures and systems. Additionally, if we are to ensure expansion and ongoing commitment to implementation science, advancing health equity will involve greater coordination with implementation science partners at federal, state, and local research organizations; community and non-governmental organizations; and health systems.

The content of this special supplement, “Advancing the Adaptability of Chronic Disease Prevention and Management Through Implementation Science,” highlights novel approaches to implementation research and practice and provides new insights on advancing chronic disease health equity. It begins an important conversation about the state of current chronic disease prevention implementation science approaches, which are critical in providing health care services—especially in the wake of the COVID-19 pandemic and subsequent shifts in public health systems—and in improving care for people at higher risk for chronic disease, including historically marginalized communities.

## Data Availability

The data presented in this study are available on request from the corresponding author.
